# Effusion into the Brain

**Published:** 1843-07

**Authors:** James George Davey


*Effusion into the Brain.*—By JAMES GEORGE DAVEY, M.D. In the spring of 1840, being then in private practice in the suburbs of London, I was hastily summoned to Johnson-street, Somerstown, to the assistance of a child, about eighteen or twenty months old, who, I was told, had been suddenly seized with a "fit." It was lying in its mother's lap, apparently dying. The surface of the body was cold, and covered with a clammy perspiration, and the countenance injected and livid; the pupils were dilated, the eyes fixed, and the eyelids partially closed; the mouth contained a quantity of frothy saliva; the respiration was hurried and oppressed; and the pulse rapid and feeble. The parents of the child, and two or three other persons who were present on my arrival, felt so assured of its immediate death, that I was requested not to interfere in any way. I had much difficulty to induce the parents to forego their resolution. Having only two or three days previously lost a child under very similar circumstances, at Camden-town, I became immediately resolved on a different kind of treatment to that I had then adopted; that is, I chose to open a branch of the temporal artery rather than wait the issue of a modified procedure. I abstracted very little short of a wineglassful of blood, and then, having desired the father to press the bleeding orifice with the point of his finger, I quickly administered, by means of a teaspoon inserted into the pharynx, a little brandy and water. The excellent effects of the treatment became immediately apparent. I was no less surprised than those about me. The surface of the body became warm, the respiration natural, and the pulse normal. My little patient was presently conscious, though much disposed to sleep. I called after four or five hours and found the child very comfortable and perfectly rational. I directed a purgative to be given. The next morning it was brought to my residence in Clarendon-square, perfectly well.

The above case, I consider highly interesting. I make no doubt that the more general practice of leeches in cases of the kind may be, in many cases, very properly substituted by general bleeding. If children are but men in miniature, wherefore should we not treat them as such? Moralists do so, and why not doctors?—*London Lancet*, April 22, 1843.

